# SRNet-YOLO: A model for detecting tiny and very tiny pests in cotton fields based on super-resolution reconstruction

**DOI:** 10.3389/fpls.2024.1416940

**Published:** 2024-08-09

**Authors:** Sen Yang, Gang Zhou, Yuwei Feng, Jiang Zhang, Zhenhong Jia

**Affiliations:** ^1^ School of Computer Science and Technology, Xinjiang University, Urumqi, China; ^2^ The Key Laboratory of Signal Detection and Processing, Xinjiang Uygur Autonomous Region, Xinjiang University, Urumqi, China

**Keywords:** cotton field, super-resolution reconstruction, feature fusion, YOLOv8, tiny pests, very tiny pests

## Abstract

**Introduction:**

Effective pest management is important during the natural growth phases of cotton in the wild. As cotton fields are infested with “tiny pests” (smaller than 32×32 pixels) and “very tiny pests” (smaller than 16×16 pixels) during growth, making it difficult for common object detection models to accurately detect and fail to make sound agricultural decisions.

**Methods:**

In this study, we proposed a framework for detecting “tiny pests” and “very tiny pests” in wild cotton fields, named SRNet-YOLO. SRNet-YOLO includes a YOLOv8 feature extraction module, a feature map super-resolution reconstruction module (FM-SR), and a fusion mechanism based on BiFormer attention (BiFormerAF). Specially, the FM-SR module is designed for the feature map level to recover the important feature in detail, in other words, this module reconstructs the P5 layer feature map into the size of the P3 layer. And then we designed the BiFormerAF module to fuse this reconstruct layer with the P3 layer, which greatly improves the detection performance. The purpose of the BiFormerAF module is to solve the problem of possible loss of feature after reconstruction. Additionally, to validate the performance of our method for “tiny pests” and “very tiny pests” detection in cotton fields, we have developed a large dataset, named Cotton-Yellow-Sticky-2023, which collected pests by yellow sticky traps.

**Results:**

Through comprehensive experimental verification, we demonstrate that our proposed framework achieves exceptional performance. Our method achieved 78.2% mAP on the “tiny pests” test result, it surpasses the performance of leading detection models such as YOLOv3, YOLOv5, YOLOv7 and YOLOv8 by 6.9%, 7.2%, 5.7% and 4.1%, respectively. Meanwhile, our results on “very tiny pests” reached 57% mAP, which are 32.2% higher than YOLOv8. To verify the generalizability of the model, our experiments on Yellow Sticky Traps (low-resolution) dataset still maintained the highest 92.8% mAP.

**Discussion:**

The above experimental results indicate that our model not only provides help in solving the problem of tiny pests in cotton fields, but also has good generalizability and can be used for the detection of tiny pests in other crops.

## 1 Introduction

Cotton ranks as one of the planet’s most crucial cash crops, serving not only as the foundational raw material for the textile industry but also offering livelihood opportunities to millions worldwide (Wang and Memon, 2020). Yet, when grown in the wild, cotton becomes vulnerable to infestations of tiny pests, leading to significant reductions in yield (Chen et al., 2020). The vast expanse of cotton fields in wild environment complicates the timely identification of these tiny pests by human observers. Often, by the time such pests are incidentally discovered, they have proliferated extensively. Thus, the prompt and effective detection and management of pests in these wild natural cotton field are imperative (Wu and Guo, 2005). In combating these frequent pest invasions, yellow sticky traps prove to be an invaluable tool (Pinto-Zevallos and Vänninen, 2013). They play a crucial role in capturing tiny pests in wild natural cotton fields, enabling timely analysis of the extent of pest damage. This facilitates swift, precise responses, including targeted pesticide application in afflicted areas. Moreover, this strategy ensures optimal use of pesticides, enhancing crop yield and ushering in an era of rapid and efficient precision agriculture.

Traditionally, pest species and quantities on sticky traps have been examined to determine the appropriateness of control measures (Aliakbarpour and Rawi, 2011). Cotton fields, often sprawling across extensive wild areas, necessitate the deployment of a substantial number of yellow sticky traps, imposing significant strain on manual inspection efforts. Moreover, manual observations carry the risk of misinterpretation, potentially leading to inappropriate pesticide application. Consequently, automated pest detection leveraging image processing (Wen and Guyer, 2012) and machine learning techniques (Silveira and Monteiro, 2009) has garnered interest among agricultural researchers (Maharlooei et al., 2017). introduced an algorithm that detects and quantifies aphids on soybean leaves through image processing techniques, yet this approach heavily relies on manually selected features, which compromises the robustness of the detector. Chunlei et al. (2012) employed shape and color characteristics derived from thresholding methods for whitefly detection following image segmentation. Xia et al. (2015) developed a methodology for the automated identification of whiteflies, aphids and thrips, employing YCbCr color attributes and a distance-based classification system. Thus, conventional pest identification methods predominantly rely on observations and identifications by agricultural experts or technicians. This approaches are not only time-consuming and costly but also sensitive to timing and challenging to apply broadly (Ye et al., 2023).

In recent years, deep learning models have demonstrated exceptional performance in pest detection (Li et al., 2021b), effectively addressing the limitations of conventional machine learning approaches (Shen et al., 2018). The motivation for designing these models mainly comes from mainstream object detection methods, which are two-stage (Girshick et al., 2014; Girshick, 2015; Ren et al., 2017) and one-stage detectors (Redmon et al., 2016; Redmon and Farhadi, 2017, Redmon and Farhadi, 2018). For instance, Patel and Bhatt (2021) utilizes Faster R-CNN for automatic multi-class pest detection in agriculture, addressing challenges of small datasets and class imbalance through image augmentation techniques like Horizontal Flip and 90 Degree Rotation. It demonstrates deep learning’s potential to significantly enhance pest detection accuracy. Tian et al. (2023) refined YOLOv3 for efficient tiny object detection, integrating this enhanced algorithm into software for pest early warning systems. Chen et al. (2022) integrated YOLOv4 into a compact vehicle equipped with a camera and auxiliary lighting to monitor pests in grain storage. Zhang et al. (2023a) substituted YOLOv7’s original optimizer with the Adan optimizer, enhancing the detection of three specific corn pests. In parallel, leveraging traps in conjunction with deep learning algorithms has proven effective in greenhouse and laboratory environment. Li et al. (2021a) conducted sticky trap data collection on greenhouse-grown pepper plants, refining Faster-RCNN for the detection of whiteflies and thrips in their adult stages, yielding promising results. Gerovichev et al. (2021) collected samples of flying insects from various locations, proposing a novel approach that combines deep learning with sticky trap-captured pests for object detection experiments using YOLOv5. Zhang et al. (2023b) devised a trap system incorporating yellow sticky traps and LED lighting, augmenting YOLOv5 with a copy-and-paste data augmentation technique for pest management in cherry tomato and strawberry greenhouses. Kalfas et al. (2023) collects data from wild chicory fields and subsequently trains the yolov5 algorithm within a laboratory environment for focused detection of two pests (chicory leaf miners and woolly aphids) and their predatory counterparts (ichneumonid wasps and grass flies). She et al. (2022) adds four attentional mechanisms to yolov5 for intelligent counting methods of cucurbits on trap bottles. Object detection algorithms utilizing deep learning play well in detecting pests in traps.

**Figure 1:**
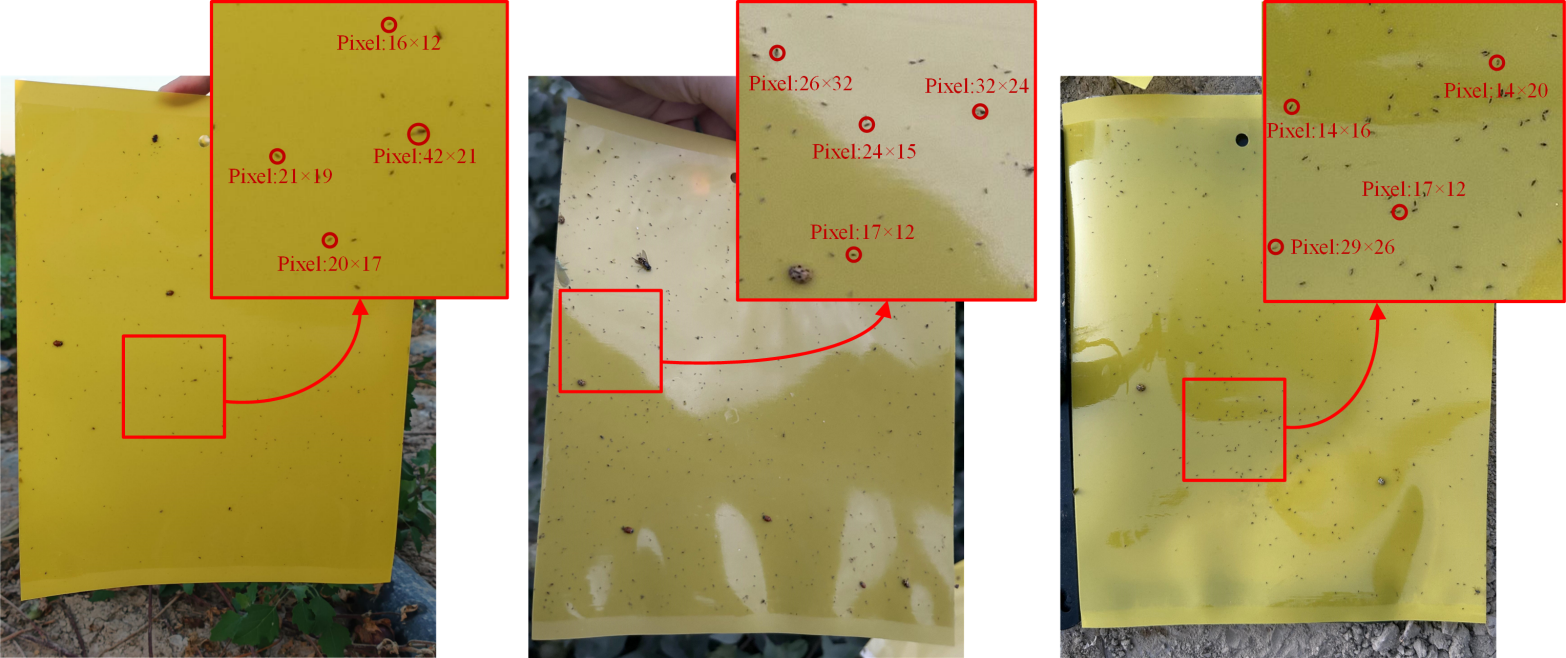
Illustration of the size of the pests. The top right image of each picture is a partial zoom image and the pixel size of some pests is labeled.

However, we found that it is difficult to achieve well results in cotton field pest detection by these methods. This is mainly due to the presence of a large number of tiny pests such as cotton thrips and Myzus-persicae. Generally, the size of the “tiny pests” is not more than 32×32 pixels on the captured image, and even “very tiny pests” are not more than 16×16 pixels, which makes it difficult to extract effective features by conventional object detection methods as shown in **Figure 1**. To address the above problems, we proposed SRNet-YOLO model for the two small-sized pests detection in cotton fields in the field. The algorithm combines super-resolution reconstruction with feature fusion to minimize false and missed detections in “tiny pest” and “very tiny pest” detection. The principal contributions of this study are outlined as follows.

1. To enhance the accuracy of detection and monitoring of tiny pest infestation in local cotton fields, we developed the Cotton-Yellow-Sticky-2023 dataset, representing yellow sticky traps in cotton fields. Additionally, we made validation experiments of the overall approach on the public Yellow Sticky Traps dataset as well.
2. The SRNet-YOLO model is proposed to specifically “tiny pests” and “very tiny pests” in wild cotton fields. The model adopted the feature extraction approach of YOLOv8 with FM-SR module and BiFormerAF module. The proposed FM-SR and BiFormerAF modules are used for feature reconstruction and feature fusion, and their joint action achieves a perfect fusion of original and recovered features to enhance the feature information of tiny and very tiny pests. These two modules enhanced the critical feature of tiny pests during feature extraction, thus improving the overall performance of the network.
3. SRNet-YOLO is trained and evaluated on both the Cotton-Yellow-Sticky-2023 dataset and the Yellow Sticky Traps datasets (low-resolution), with its better performance compared against other advanced object detection algorithms.

## 2 Related work

### 2.1 Object detection model

In the domain of computer vision, the two-stage object detection RCNN model, grounded in CNN technology, represents pioneering efforts to integrate deep learning into object detection. This model employs region proposal mechanisms to identify potential regions of interest, subsequently processing each region through a CNN for feature extraction. This approach enables precise identification of object categories within each region and facilitates the regression adjustment of their boundaries (Girshick et al., 2014). Throughout the evolution of two-stage target detection, enhancements to the RCNN framework, including Fast-RCNN (Girshick, 2015), Faster-RCNN (Ren et al., 2017), and Mask-RCNN (He et al., 2017), have yielded improved accuracy. Jiao et al. (2020) proposed an unanchored regional convolutional neural network, AF-RCNN, consisting of an unanchored regional proposal network, AFRPN, and a Faster R-CNN end-to-end for the detection of 24 classes of pests. This method improves accuracy, but they are processing images at 0.07 seconds a photo, which is still some distance away from 30FPS detection. This is usually where second-stage object detection falls short as well, which makes it difficult to make timely agricultural decisions after detecting “tiny pests” and “very tiny pests” when dealing with large amounts of data.

In the realm of agricultural pest identification and detection, it’s essential for the object detection system to achieve precise recognition alongside real-time detection capabilities. Given the rapid development, enhanced accuracy, and reduced parameterization of the YOLO series of CNN-based one-stage object detection models, they emerge as highly suitable for practical agricultural pest detection applications. The inception of the YOLO series was marked by YOLOv1 (Redmon et al., 2016), the pioneering one-stage end-to-end object detection algorithm that approached object detection as a regression problem. By processing the entire image as input, it facilitated the generation of bounding boxes through a neural network, significantly accelerating detection speeds compared to two-stage methods. YOLOv3 (Redmon and Farhadi, 2018) further refined the detection mechanism by replacing the original Darknet19 with Darknet53 and adopting a pyramid network structure for multi-scale detection. Its shift to logistic regression for classification, over softmax, balanced real-time performance with detection accuracy. Lv et al. (2022) proposed a linear transformation method based on K-means and alteration of the original yolov3 framework to improve the detection of corn pests. YOLOv4 (Bochkovskiy et al., 2020) enhanced the architecture by integrating Darknet53 with CSPNet, introducing residual blocks for improvements, and adding the SPP module to expand the receptive field without compromising speed. Liu et al. (2022) proposed a fusion of triplet attention mechanism with yolov4 and introduced a focus function to solve the tomato pests sample imbalance problem. YOLOv5 (Jocher et al., 2022) innovates with the mosaic data augmentation method and a focus structure within the backbone, enriching object backgrounds while optimizing the detection of small object and reducing computational demands. Huang et al. (2023) introduced the attention module (CBMA) and adaptive spatial feature fusion (ASFF) to yolov5 to improve the detection of *Laodelphax striatellus*. Addressing the issue of over-optimization in YOLOv4 and YOLOv5, YOLOX (Ge et al., 2021) reverts to YOLOv3 and Darknet53 as foundational models, incorporating enhancements like an increased EMA weight update and transitioning from a coupled to a decoupled header to significantly boost convergence speed. Additionally, it adopts an anchorless mechanism, reducing parameter count to enhance detection capabilities. Xu et al. (2023) proposed an adaptive spatial feature fusion and lightweight detection model (ASFL-YOLOX) of positional IPPs for pests of Papilionidae (IPPs), which is much better than Faster R-CNN in both map and inference. The creators of YOLOv4 and Scaled-YOLOv4 (Wang et al., 2021) unveiled the YOLOv7 (Wang et al., 2023) algorithm. This latest iteration integrates model reparameterization into its architecture, along with an augmented efficient layer aggregation network and a variety of trainable “bag-of-freebies” techniques. These innovations aim to improve the accuracy of network training and detection without elevating inference costs. Jia et al. (2023) utilized MobileNetV3 for feature extraction and combined CA attention and SIoU loss function to improve yolov7, which plays a better role in the detection of rice pests and diseases. One-stage object detection has the advantages of being fast and easy to deploy, and is favored by researchers in smart agriculture. However, for the study of “tiny pests”, especially “very tiny pests”, the localization function of the one-stage object detection model is a bit poor.

Both two-stage and one-stage common object detection are less often applied to “tiny pests” and “very tiny pests”. The main reason is that the pests are too small, the lower resolution leads to fewer features, and the process of extracting features is more difficult. For one-stage although results can be made quickly, the accuracy of this result may be questionable. And the two-stage is the opposite. Therefore, we designed a new model applied to the detection of “tiny pests” and “very tiny pests” in cotton fields, which model maintains the advantages of the one-stage approach while having higher detection results.

### 2.2 Tiny pests research

Small object detection is crucial for precise pest identification, crop protection, and minimizing pest-induced harm (Du et al., 2022). Due to small objects containing fewer pixels, their features are less distinct (He et al., 2016; Xie et al., 2017; Gao et al., 2021), apply downsampling to feature maps to reduce resolution, aiming to decrease spatial redundancy and learn high-dimensional features. However, this approach risks losing object information. Studies focused solely on detecting tiny pests in smart agriculture are notably limited. Li et al. (2022) applies a spectral residual model and a support vector machine to effectively detect tiny pests in trapping images, achieving high accuracy. This method offers a time-efficient alternative to manual counting for pest management. Wen et al. (2022) introduced Pest-YOLO, a novel agricultural pest detection model using focal loss and non-IoU box strategies for improved accuracy on dense and tiny pests. Validated on the Pest24 (Wang et al., 2020) dataset, it demonstrates superior performance over other models, indicating its efficacy for practical pest monitoring. Wang et al. (2022) presents S-ResNet, an improved version of the standard ResNet model tailored for the identification of tiny pests, by optimizing its structure and integrating advanced modules for enhanced feature processing. This novel neural model has shown to surpass the traditional ResNet in terms of performance, indicating its potential utility in agricultural pest detection applications. Wang et al. (2023) presents an auxiliary prior-knowledge architecture for tiny pests detection in wild environments, capable of identifying rarely collected pests with minimal sample availability. Liu et al. (2023) enhances DETR’s ability to detect small objects important for forest pest control by adding skip connections and spatial pyramid pooling, significantly improving detection precision.

Although research on tiny pests has progressively advanced in agriculture, most of it has been conducted on “tiny pests” around 32×32 pixels, and very few models have been developed for “very tiny pests” below 16×16 pixels. Because many “very tiny pests” do exist in cotton and are not easy to detect. Therefore we created a “tiny pest” and “very tiny pest” dataset containing cotton fields named Cotton-Yellow-Stricky-2023. This dataset will be used for experiments with our new model.

## 3 Materials

### 3.1 Image data acquisition and annotation

**Figure 2:**
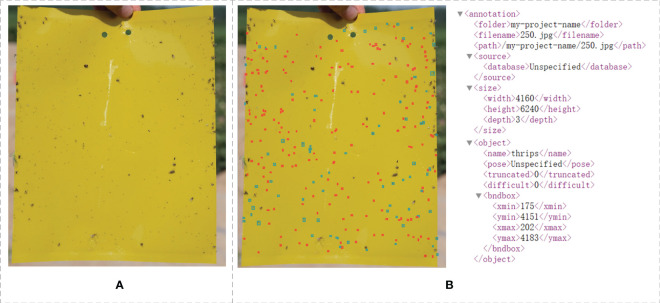
Example pictures of yellow sticky traps taken in a cotton field Original **(A)** and labeled **(B)**.

Owing to the lack of a dataset capturing yellow sticky traps in naturally wild cotton fields, this research utilized a dataset derived from yellow sticky traps collected in an experimental cotton field from Huaxing Form, Urumqi City, Xinjiang Province, which is located in the environment of 86.9°E and 44.23°N. The collection period was strategically chosen to coincide with the peak cotton pest season, spanning from early July to early September 2023. As cotton will be infested by thrips, aphids and whiteflies during the growth period, and these pests are sensitive to yellow color and have a strong tendency to yellow, so in the process of data collection, we chose the yellow sticky traps for the collection of pests. For the collection methodology, clean yellow sticky traps were strategically placed at 20-meter intervals across the cotton field and were collected and photographed every 4 hours to ensure a broad generalization of the model. To capture the data, three distinct smartphones were utilized (iPhone 12, Glory 9 Android, and Xiaomi 11 Android), resulting in a total of 387 high-resolution images. The primary focus of the research was on two classes of “tiny pests” and “very tiny pests”: cotton thrips and Myzus-persicae, as shown in **Figure 2A**.

**Figure 3:**
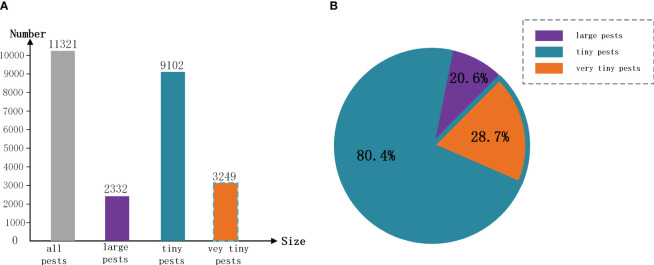
Schematic representation of the number and percentage of “tiny pests” and “very tiny pests”. Where tiny pests are contained “very tiny pests”, “large pests” are pests with pixels larger than 32 × 32 pixels. **(A)** is a histogram of the number of pests, **(B)** showing the percentage of pests.

The process of image object labeling is pivotal in deep learning, highlighting the significance of a standardized selection of labeling tools. In this study, we utilized the LabelImg tool to annotate the presence and species of pests on yellow sticky traps. Upon completion of the labeling task, an XML file corresponding to each image’s name is created, encapsulating the image’s dimensions, the label names, and their positions as shown in **Figure 2B**. For subsequent experimental directions, a detailed analysis of the annotated dataset revealed that objects with dimensions smaller than 32×32 pixels (contained 16×16 pixels) constitute 80.4% of all labeled pests, underscoring the prevalence of “tiny pests” within these images. Notably, objects smaller than 16×16 pixels account for 28.7% of all labeled pests, categorizing them as “very tiny pests”. Detailed quantities and weights are shown in **Figure 3**. In terms of the percentage and size of these pests, from our human eyes, some tiny pests are difficult to distinguish.

### 3.2 Dataset preparation

**Figure 4:**
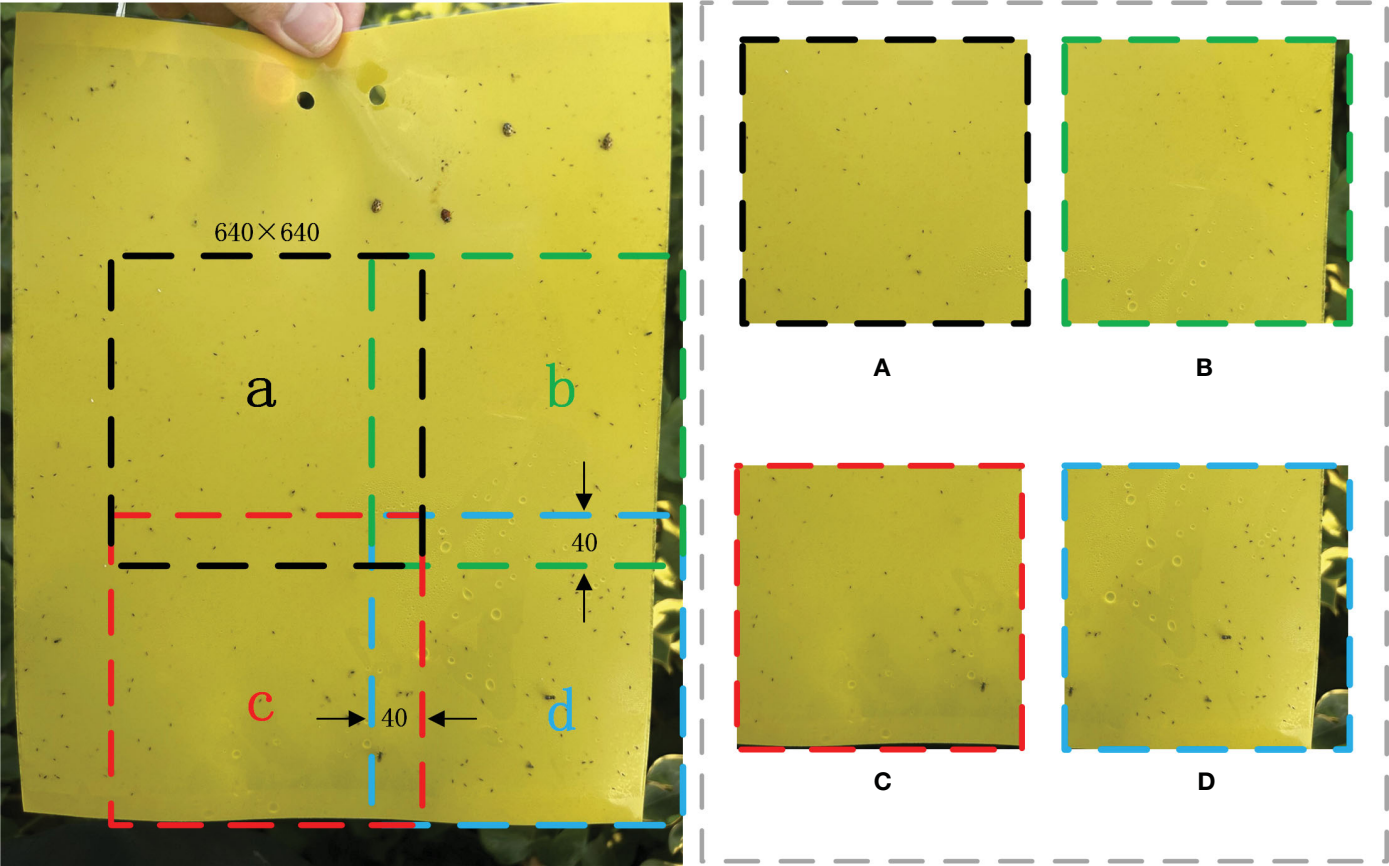
Segmented image approach example image, where **(A-D)** are cropped image data.

This study employs a segmented image approach to generate the dataset for experimental purposes. Images are cropped to a dimension of 640×640 pixels, ensuring a 40-pixel overlap between adjacent cropped sections to prevent the loss of objects subjects during the cropping process. This method is illustrated schematically in **Figure 4**. We chose  640 × 640 pixels because this is among the optimal inputs for most of the models, and the reason for overlapping two adjacent sheets by 40 pixels is that we counted all pest targets up to a maximum of 40 pixels, so overlapping them by 40 pixels would leave all targets intact in the segmented image. The resulting dataset, designated “Cotton-Yellow-Sticky-2023”. Our dataset consists of a training set (all pests with pixels in the data are randomly selected), a test A (all pests with pixels are randomly selected), a test B (all pests with pixels smaller than 32 × 32 pixels, which is the “tiny pests” test set), and a test C (all pests with pixels smaller than 16 × 16  pixels, which is the “very tiny pests” test set), as shown in **Table 1**. In test A, we have a data count of pest targets, which results in 78% of tiny pests smaller than 32 × 32  pixels, this averages out to the presence of at most one pest object larger than  32 × 32 pixels per image. test B is from test A, and our construction rule is to keep only the data images where all the pixels of the pest targets are smaller than 32 × 32  or less. Thus test B is an extreme case of test A. The data in test C is different from test A. The selection rule of test C is similar to that of test B. Only the data images with all pest pixels less than 16×16 are selected.

**Table 1:** Distribution of data sets.

Data Grouping	train	val	test		
Cotton-Yellow-Sticky-2023	1462	342	test A	test B	test C
			356	6	56
The Yellow Stricky Traps (Low resolution)	1237	300	test D: 309		

To verify the effectiveness of our model on other tiny pests detection, we chose a publicly available dataset the Yellow Sticky Traps dataset (Deserno and Briassouli, 2021), and reduced the images to obtain tiny pest samples. In order to prevent damage to the image quality after reducing the image pixels, we reduce the jagged effect in this process, which makes the output image smoother and maximizes the image quality. Specifically, we reduced the average pixel size of the NC (Nesidiocoris) from 130×130 to 31×31 , the MR (Macrolophus) from 135×121 to 33×32 , and the WF (Whiteflies) from 50×47 to 13×12 . Overall, the average pixel size of all objects was reduced from 105×100 to 25×24 . This modified dataset is called “the Yellow Sticky Traps dataset (low-resolution)” and is used as the second experimental dataset for the subsequent analysis, where the test set is named test D, as shown in **Table 1**.

## 4 Methods

### 4.1 Network structure of the SRNet-YOLO

**Figure 5:**
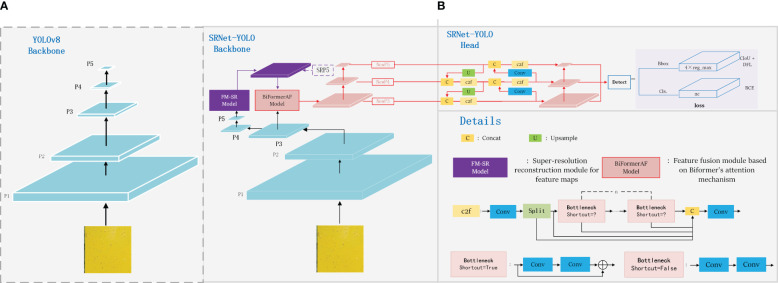
SRNet-YOLO model structure. In order to highlight the improvement of SRNet-YOLO, where **(A)** shows the feature map extraction process of backbone for YOLOv8, and **(B)** shows the feature map extraction process and detection process of backbone for SRNet-YOLO.

**Figure 6:**
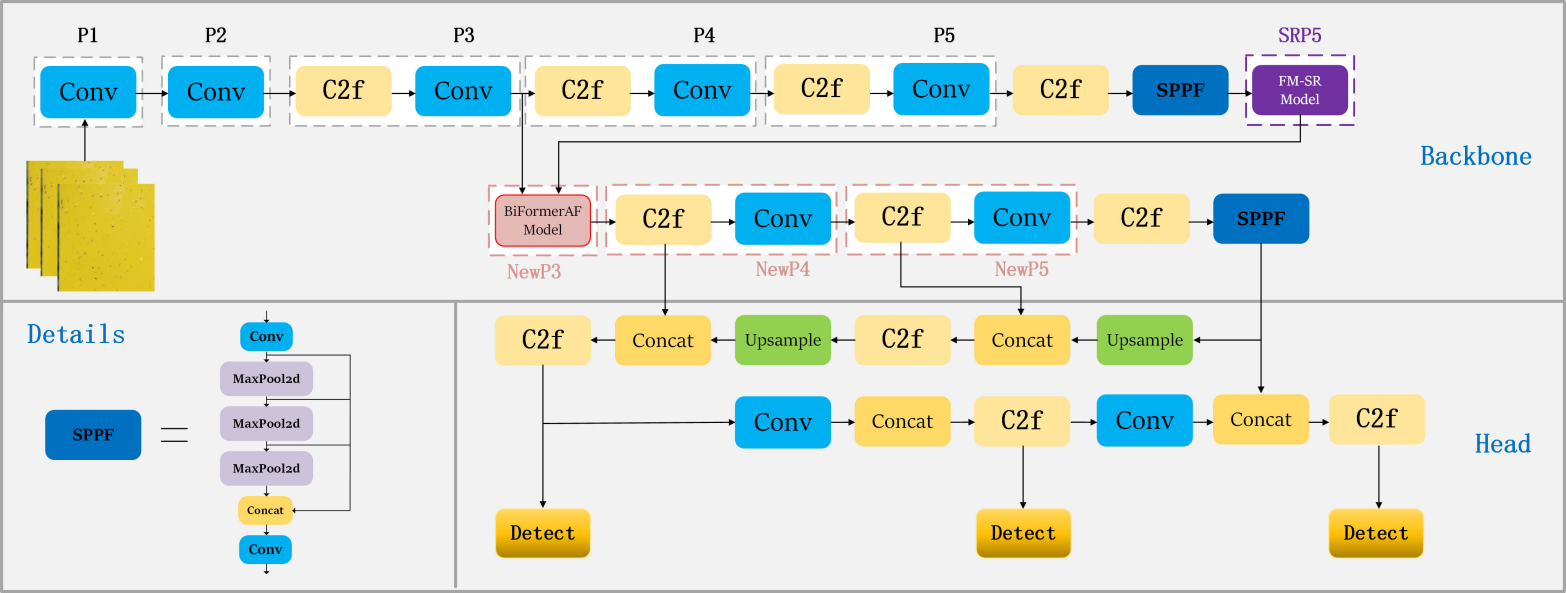
Detailed flowchart of the SRNet-YOLO model.

In this study, we proposed the SRNet-YOLO model as shown in **Figure 5B**. Our approach uses YOLOv8’s backbone (Terven and Cordova-Esparza, 2023) as a baseline, as shown in **Figure 5A**, YOLOv8 maintains the core of the CSP (Cross-Stage Partial Network) concept while designing the C2f module, which allows to improve the efficiency of the model without compromising its robustness, thus achieving an optimal balance between accuracy and computational speed. However, YOLOv8 performs poorly on small (especially very small) objects, so we improved the backbone in a more targeted way. The backbone of SRNet-YOLO contains the FM-SR and BiformerAF modules, the combination of which is very effective in improving feature extraction. In YOLOv8, the P5 layer information basically contains only the more comprehensive key information layer, and the corresponding P3 layer feature map size is 80×80, which is used for detecting targets with pixels larger than 8×8, and is applicable to the detection of small targets. So we reconstruct the P5 layer feature map by super-resolution through the FM-SR module to restore it to a dimension comparable to the P3 layer feature map, and obtain the SRP5 layer feature map. Subsequently, the SRP5 and P3 layer feature maps are jointly fused by BiformerAF to form the NewP3 feature map. The NewP3 feature map here replaces the original P3 layer feature map in YOLOv8, and this improvement solves the problem of details that may be lost during the recovery phase and enhances the functionality of the P3 layer. Subsequent feature extraction of NewP3 yields NewP4 and NewP5, which correspondingly replace the original P4 and P5 layers in YOLOv8. Above is the feature map extraction flowchart in backbone, meanwhile, we provide the detailed flowchart of the model of SRNet-YOLO as shown in **Figure 6**.

### 4.2 Feature map super-resolution reconstruction

Upsampling typically depends on a fixed interpolation algorithm that lacks adaptability and exhibits consistent performance across images. Unlike upsampling, super-resolution reconstruction provides a more advanced capability for image enhancement compared to simple upsampling methods, making it the preferred choice in practical applications. Super-resolution reconstruction is a critical research challenge in the fields of computer science and image processing. This image processing technique (Yang et al., 2014) aims to produce high-resolution images from their low-resolution versions, crucial for improving image quality, enhancing detail reproduction, and boosting image performance in a variety of applications. With the advent of deep learning and convolutional neural networks, exemplified by SRCNN (Dong et al., 2016), the first deep learning model for supersampling, super-resolution reconstruction can now effectively learn and interpret contextual information within images, significantly increasing image accuracy at the pixel level.

**Figure 7:**
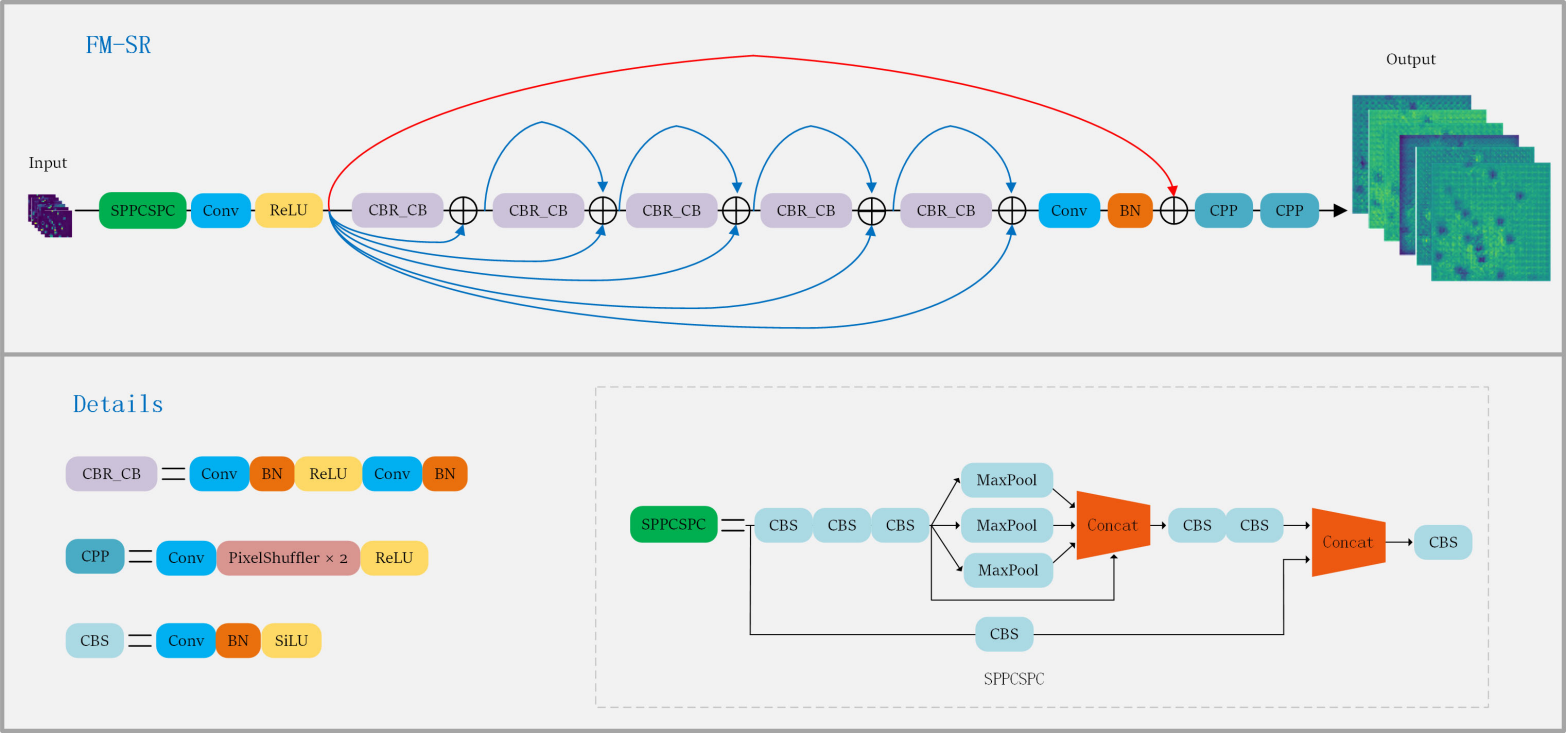
FM-SR and SPPCSP structures.

In this study, we proposed a super-resolution reconstruction module (FM-SR) dedicated to feature map enhancement, inspired by SRResNet (Ledig et al., 2017) and SPPCSPC (Wang et al., 2023), as shown in **Figure 7**. We designed FM-SM to improve on SRResNet, but with the difference that FM-SR is better used at the feature map level, while SRResNet is used at the image level. SRResNet primarily comprises five ResNet blocks, serving as the generative networks within SRGAN. SPPCSPC module as shown in **Figure 7**, it contains SPP (He et al., 2015) module and CSP module. Among them, SPP acts after maxpooling operation of 1×1 , 5×5 , 9×9 and 13×13 sized convolutional kernels as a way to obtain four different receptive fields, which are used to differentiate between large and small objects. The features will be processed through two parts of CSP module, one part consists of Conv, Batch normalization and SilU activation functions, the other part is SPP, finally the two parts of features are spliced together by concat. These operations will greatly improve the detection accuracy. Our approach is to add the SPPCSPC module to the input of SRResNet, as well as the jump connections operation (He et al., 2016), which not only enriches the deep features of the network, but also enhances the recovery of the feature images. In the whole process of FM-SR, the SPPCSPC at the input end obtains different receptive fields at the feature map level and sends them to SRResNet, which has the advantage of improving the grasp of the key information of the feature maps, while the subsequent jump connection serves to prevent the possibility of information loss after entering SRResNet.

### 4.3 Attention Fusion Mechanisms (BiformerAF)

**Figure 8:**
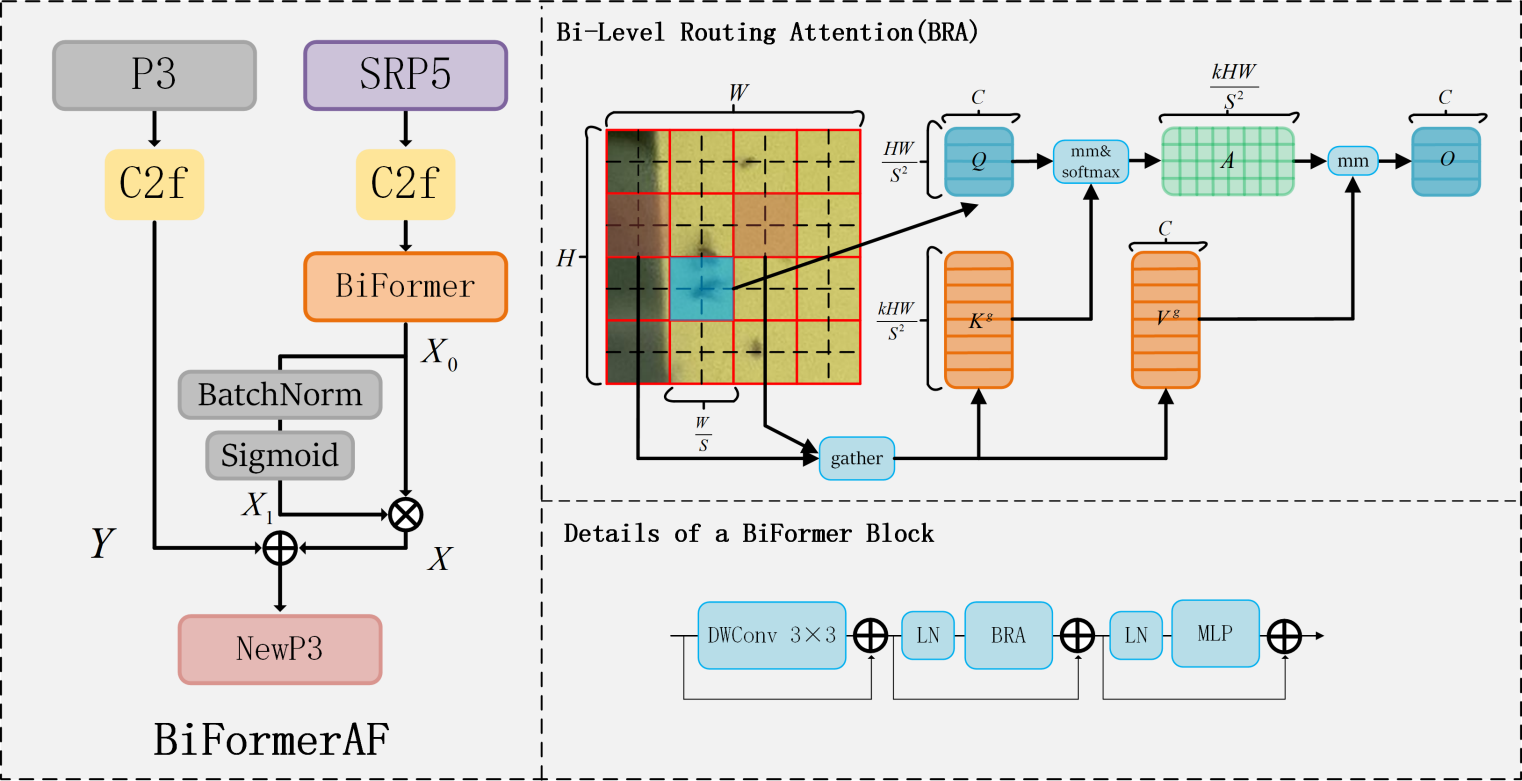
BiFormerAF, BRA and BiFormer structures.

Attention mechanisms are widely utilized in computer science for rapid and efficient analysis of complex information, significantly improving the performance of numerous deep learning architectures. BiFormer (Zhu et al., 2023) introduces a dynamic, query-aware sparse attention mechanism, predicated on the Bi-Level Routing Attention (BRA) module. The BRA module and details of a BiFormer block are shown in **Figure 8**, focusing on eliminating the majority of irrelevant key-value pairs at a coarse area level to retain a select segment of the routing area. The BRA module is structured around three principal components: region partition and input projection, region-to-region routing with directed graph and token-to-token attention.

#### 4.3.1 Region partition and input projection

Firstly, the feature map $X\in R^{H\times W\times C}$ is divided into S×S non-overlapping regions, each containing $\frac{HW}{S^{2}}$ feature vectors, X becomes $X^{r}\in R^{S^{2}\times \frac{HW}{S^{2}}\times C}$ , and then $Q,K,V\in R^{S^{2}\times \frac{HW}{S^{2}}\times C}$ is obtained by linear mapping with the expression as Equation 1.


(1) $$\begin{array}{c} Q=X^{r}W^{q},K=X^{r}W^{k},V=X^{r}W^{v} \end{array}$$

where $W^{q},\;W^{k},\;W^{v}\in R^{C\times C}$ is the projection weight of query, key, and value, respectively.

#### 4.3.2 Region-to-region routing with directed graph

A directed graph is constructed to find the regions that should be involved without a given region. First calculate the average of Q and V in each region to get $Q^{r},K^{r}\in R^{S^{2}\times C}$ . We then derive the adjacency matrix $A^{r}\in R^{S^{2}\times S^{2}}$ , of region-to-region affinity graph via matrix multiplication between $Q^{r}$ and $K^{r}$ , as Equation 2. Entries in the adjacency matrix, $A^{r}$ , measure how much two regions are semantically related. Then modify the correlation graph by keeping only the first K connections of each region, have routing index matrix $I^{r}\in N^{S^{2}\times K}$ , and save the indexes of the first K connections row by row, as Equation 3.


(2) $$\begin{array}{c} A^{r}=Q^{r}{\left(K^{r}\right)}^{T} \end{array}$$

(3) $$\begin{array}{c} I^{r}=topkIndex\left({\text{A}}^{r}\right) \end{array}$$

where the $i^{th}$ row of $I^{r}$ contains the indexes of the first k most relevant regions of the $i^{th}$ region.

#### 4.3.3 Token-to-token attention

For each Query token in each region i , it will focus on all key-value pairs in the concatenation set of the K routing regions, indexed $I_{\left(i,1\right)}^{r},I_{\left(i,2\right)}^{r},\cdots ,I_{\left(i,k\right)}^{r}$ . According to the set we first aggregate the key and value tensor as Equation 4.


(4) $$\begin{array}{c} K^{g}=gather\left(K,I^{r}\right),V^{g}=gather\left(V,I^{r}\right) \end{array}$$

where $K^{g},\;\;V^{g}\in R^{S^{2}\times \frac{kHW}{S^{2}}\times C}$ are the tensor of key and value after aggregation and then attention operation is used on the aggregated K-V pairs as Equation 5.


(5) $$\begin{array}{c} O=Attenion\left(Q,K^{g},V^{g}\right)+LCE\left(V\right) \end{array}$$

where, a local context augmentation term LCE(V) (Ren et al., 2022) is introduced, function LCE(·) is parameterized with a depth-wise convolution, and we set the convolution kernel size to 5.

He et al. (2024) added BiFormer to YOLOv7 to improve the detection of tea pests. They only added BiFormer behind the feature maps of P3, P4 and P5 layers in Neck, but not in the process of feature extraction, and it will have the potential for the loss of critical information. The difference is that we designed a fusion mechanism based on BiFormer to be added into the backbone, which acts on the fusion of the SRP5 and P3 feature maps for more accurate feature extraction in the follow-up, thus improving the performance of the model.

In this paper, we develop a BiFormer-based feature fusion mechanism, called BiFormerAF, for fusing feature mappings between the P3 and SRP5 layers in feature extraction, as shown in **Figure 8**. The mechanism is divided into two branches: one processes the P3 layer feature image through the C2f module, producing an output denoted as Y , as described in Equation 6. The other branch processes the SRP5 layer feature map, which is the P5 layer feature image enlarged fourfold by FM-SR module, through the C2f module. Subsequently, most irrelevant key-value pairs are filtered out using BiFormer to eliminate redundancy, with the filtered output labeled $X_{0}$ , as described in Equation 7. It is important to note that the $X_{0}$ is then passed through BatchNorm and Sigmoid operations sequentially, generating the output $X_{1}$ , as described in Equation 8. $X_{1}$ serves as weights assigned to $X_{0}$ , facilitating their element-wise multiplication to obtain new information, X , as described in Equation 9. Finally, the information Y and X are merged through element-wise addition, as described in Equation 10, with the combined output forming the updated information for the NewP3 layer.


(6) $$\begin{array}{c} Y=C2f\left(P3\right) \end{array}$$

(7) $$\begin{array}{c} X_{0}=BiFormer\left(C2f\left(SRP5\right)\right) \end{array}$$

(8) $$\begin{array}{c} X_{1}=Sigmoid\left(BatchNorm\left(X_{0}\right)\right) \end{array}$$

(9) $$\begin{array}{c} X=X_{0}\times X_{1} \end{array}$$

(10) $$\begin{array}{c} NewP3=Y+X \end{array}$$

In C2f, firstly the convolution operation helps to extract features of different levels and abstraction degrees from the input data, secondly the branching design of c2f helps to increase the nonlinear and representation capabilities of the network, which improves the network’s ability to model complex data, and finally after feature splicing enriches the feature expression capability. The output which is subsequently processed by Biformer is two branches, one of which is used to multiply the weight factors with the other to highlight important features. Since the output values of Biformer may have a large distribution, and the span is too large to use sigmoid directly, we first use the BN layer as an intermediate buffer. The BN layer can make the distribution of features more stable by normalizing the data of each mini-batch, so that the mean and variance of these features are changed to 0 and 1, respectively. And the BN layer can also accelerate convergence and prevent gradient explosion, so the BN layer plays a good effect in the middle. Finally the input is changed to between (0, 1) using sigmoid to multiply with another branch. Finally feature fusion according to the addition of elements (add) is performed, which increases the amount of information under the features describing the image, but the number of dimensions describing the image itself does not increase, only the amount of information under each dimension, which is clearly beneficial for the detection of the final image.

Therefore, we perform super-resolution reconstruction work at the feature extraction P5 layer, which allows us to magnify on the key information to form the SRP5 layer, which helps the subsequent feature extraction. We let the SRP5 information target “tiny pests” and “very tiny pests” information effectively by BiFormer attention in a query self-attention manner. After that we did normalization as weights multiplied with itself for highlighting the weight of feature information. We consider the possibility that there is information that has not been extracted before the super-resolution, so we perform feature fusion of the SRP5 information with the P3 information to prevent the information from being missed.

### 4.4 Loss function

We use the FM-SR modules of the holistic framework for end-to-end training, ensuring that the new modules designed are adapted to each other and that the loss function is computed at the detection output of the model. Our aim is for the final detection task, and not adding the pre-training weights of FM-SR and thus not being limited by the weights is a better choice for the final accurate detection. We do this with the benefit of being more adaptable to a specific task and having flexibility. Moreover, we can adjust and optimize our model based on the performance during the training process in an end-to-end manner. In this study, we still take the CIoU loss function (Zheng et al., 2022), shown in Equation 11.


(11) $$\begin{array}{c} L_{cIoU}=1-IoU+\left(\frac{\rho^{2}\left(b,\;b^{gt}\right)}{c^{2}}\right)+\alpha v \end{array}$$

where α is the weight parameter. v is the similarity used to measure the aspect ratio. $\rho^{2}$ denotes the Euclidean squared distance between the two held bounding boxes. $c^{2}$ denotes the square of the diagonal distance between the two rectangular boxes. b and $b^{gt}$ denote the centroids of the two bounding boxes. CIoU takes into account overlap area, centroid distance and aspect ratio and has better regression accuracy.

## 5 Experimental results

Utilizing our specially compiled dataset, Cotton-Yellow-Sticky-2023, we conducted comprehensive experiments to assess the superiority of our proposed method by comparing it with several cutting-edge approaches. Moreover, we validated the effectiveness of our method by testing on the Yellow Sticky Traps dataset (low-resolution).

### 5.1 Experimental setup

All experiments were conducted on an NVIDIA RTX 3090 GPU, which boasts 24 GB of RAM. The development environment employed was PyCharm 2021.3, alongside the programming language Python 3.8. For deep learning tasks, we utilized the frameworks Torch 1.10.0 and torchvision 0.11.1. Image processing was facilitated through the use of OpenCV, and CUDA 11.3 served as the acceleration environment. This research builds upon enhancements to the YOLOv8 network model, employing yolov8n object detection weights for training purposes. The detailed parameters are presented in **Table 2**.

**Table 2:** Hyperparameters for network training setts.

Hyperparameter	Value
Epoch	1000
Batch Size	32
Image Size	640
Seed	0
Ir0	0.001
Irf	0.01
Pretrained Model Weights	yolov8n.pt

To thoroughly evaluate the effectiveness of our model, we conducted a series of experiments on both our proprietary dataset and a public dataset after reducing its pixel resolution. We utilized the subsequent quality metrics for assessment: Precision (P), Recall (R), Mean Average Precision (mAP), and F1-score (F1). These metrics are mathematically described as Equations 12–16.


(12) $$\begin{array}{c} \text{Precision}=\frac{\text{TP}}{\text{TP}+\text{FP}} \end{array}$$

(13) $$\begin{array}{c} \text{Recall}=\frac{\text{TP}}{\text{FN}+\text{TP}} \end{array}$$

(14) $$\begin{array}{c} \text{F}1-\text{score}=\frac{2\text{TP}}{2\text{TP}+\text{FN}+\text{FP}} \end{array}$$

(15) $$\begin{array}{c} \text{AP}=\int_{0}^{1}\text{P}\left(\text{R}\right)\text{dR} \end{array}$$

(16) $$\begin{array}{c} \text{mAP}=\frac{1}{N}\sum_{1}^{N}AP \end{array}$$

where TP, TN, FP, and FN denote true positive, true negative, false positive, and false negative, respectively. Accuracy is the proportion of correctly predicted observations to the total observations. P (Precision), also known as positive predictive value, is the ratio of true positives to the sum of true positives and false positives, indicating the correctness of positive predictions. R (Recall), or sensitivity, measures the ratio of true positives to the sum of true positives and false negatives, reflecting the model’s ability to identify all relevant instances. AP (Average Precision) refers to individual category average precision, while mAP (Mean Average Precision) is the average precision of all class. The F1-score is the harmonic mean of precision and recall, providing a balance between them for a comprehensive measure of the model’s accuracy.

### 5.2 Comparison of different object detection algorithms

In our study, we meticulously compared several object detection algorithms to assess their efficacy in identifying tiny pests. The evaluation utilized the “Cotton-Yellow-Sticky-2023 test A” dataset, aiming to provide a detailed analysis of each algorithm’s performance. The algorithms comparison includes Faster R-CNN, YOLOv3, YOLOv5, YOLOv7, YOLOv8, YOLOv9 and our proposed model (SRNet-YOLO). The evaluation metrics used for comparison were P, R, mAP, and F1, as these metrics provide a holistic view of the model’s accuracy, reliability, and efficiency in tiny pest detection. The results of the comparison are shown in **Table 3**, reveal significant differences in the performance of the algorithms. Faster R-CNN demonstrated the lowest precision at 42.8% and a recall of 65.3%, leading to a mAP of 44.6% and an F1 of 0.52. This indicates a considerable gap in detecting tiny pests accurately. The advancements in YOLO algorithms, particularly YOLOv3, YOLOv5, YOLOv7, YOLOv8 and YOLOv9, have significantly enhanced tiny pests detection performance, among them, YOLOv9 achieved the highest precision of 75.1%, and YOLOv8 achieved the highest recall of 73.2%, the highest mAP of 74.1% and F1-score of 0.73. These improvements underscore the efficiency of YOLO models in processing and analyzing images, outperforming previous versions by maintaining high precision and recall rates.

**Table 3:** Detection results of different object detection algorithms in Cotton-Yellow-Stricky-2023 test A.

Model	Precision (%) ↑	Recall (%) ↑	mAP (%) ↑	F1-score
Faster R-CNN	42.8	65.3	44.6	0.52
YOLOv3	69.7	72.5	71.3	0.71
YOLOv5	67.7	76.4	71.0	0.72
YOLOv7	70.4	77	72.5	0.73
YOLOv8	73.3	73.2	74.1	0.73
YOLOv9	75.1	67.8	72.6	0.71
SRNet-YOLO(ours)	**75.5**	**78.1**	**78.2**	**0.77**

The bold value means the highest value, and the symbol (↑) means the higher value is better.

Our proposed model surpassed all the compared algorithms by achieving the highest P of 75.5%, R of 78.1%, mAP of 78.2%, and F1 of 0.77. This is made possible by super-resolution reconstruction techniques and feature fusion mechanisms. We design FM-SR which can be better applied to feature map level recovery and has a better grasp of the recovery of key information of feature maps. Then the more accurate recovered information is fused with the P3 layer feature map by BiformerAF to refine the P3 layer information and form NewP3. The subsequent feature extraction greatly increases our detection results, and this idea also largely solves the problem of the loss of information about the tiny pest targets during the feature extraction process. Therefore, for tiny pest object detection, SRResNet-YOLO can stand out among the advanced algorithms.

### 5.3 Ablation experiment

According to **Table 4**, our ablation study meticulously evaluated the contributions of SRResNet, FM-SR, ADD and BiFormerAF within the SRNet-YOLO framework. Where ADD is the fusion of feature map information according to the summation of corresponding elements. The purpose of our ablation experiments is to verify that FM-SR is better for feature graph level recovery compared to SRResNet and that BiformerAF has better fusion capability compared to ADD. It is also demonstrated that the combination of FM-SR and BiformerAF is the most effective. To ensure the correctness of the ablation experiments, the model hyperparameter settings and runtime environment were kept consistent, and the dataset used was cotton-yellow-sticky-2023 test A.

**Table 4:** Results of ablation experiments.

Yolov8	SRResNet	FM-SR	Add	BiFormerAF	P (%) ↑	R(%) ↑	mAP (%) ↑	F1 ↑
**✓**	**-**	**-**	**-**	**-**	73.3	73.2	74.1	0.73
**✓**	**✓**	**-**	**-**	**-**	70.3	74.2	72.9	0.72
**✓**	**✓**	**-**	**✓**	**-**	71.3	77.4	73.3	0.74
**✓**	**✓**	**-**	**-**	**✓**	73.7	74.7	75.6	0.74
**✓**	**-**	**✓**	**-**	**-**	**76**	73.9	7.5	0.75
**✓**	**-**	**✓**	**✓**	**-**	75.7	75.1	75.5	0.75
**✓**	**-**	**✓**	**-**	**✓**	75.5	**78.1**	**78.2**	**0.77**

✓ represents the use of this module in the model.

The bold value means the highest value, the symbol (↑) means the higher value is better and (-) represents a space.

From the experimental results related to SRResNet, it can be seen that R is improved in the case of using only SRResNet, which shows that the super-resolution reconstruction can prevent the loss of tiny pest targets. When ADD fusion is performed with the original P3 layer, all the metrics are improved compared to using only SRResNet, which shows that the fusion effect makes the combination of original and recovered features can improve the detection results of the model. After replacing the ADD fusion with our BiformerAF fusion, all results are greatly improved and all exceed the baseline. This shows that we chose the right attention mechanism and that multiplying the over-scored features as weight factors can highlight key information and better improve the refinement of the information, but the precision did not improve much.

To improve the precision, we performed the same experiment using FM-SR. We found that the highest accuracy of 76% was achieved when using only FM-SR, this also proves the effectiveness of recovery of feature maps by FM-SR in detecting “tiny pests” and “very tiny pests”, but R and mAP were not as good as the combination of SRResNet and BiformerAF, which was not the result we wanted. Therefore, we take that the combination of FM-SR and BiformerAF will improve this situation. From the experimental results, we can see that all the results after the combination of FM-SR and BiformerAF are the best, P, R and mAP and are higher than the baseline by 2.2%, 4.9% and 4.1%, respectively.

Therefore, the recovery of our proposed FM-SR at the feature map level is stronger than SRResNet, and BiformerAF is also superior to the ADD fusion approach. Similarly, SRNet-YOLO performed the best. The results of the ablation study clearly demonstrate the joint influence of each component on the detection effectiveness of SRNet-YOLO, which greatly improves the accurate identification of tiny pests.

### 5.4 Comparison of tests for “tiny pests” and “very tiny pests”

To verify the performance of our method on “tiny pest” and “very tiny pest”, we specifically tested it on two types of size pests, as shown in **Table 5**. In our “tiny pests” test B, our model test results were optimal and each broke new heights, with P, R, mAP and F1 breaking 70%, 90%, 90% and 0.8 for the first time to 71.1%, 95.8%, 92.4% and 0.82, outperforming each of the second-highest metrics by 5.3%, 6.2%, 11.8% and 0.06. This illustrates our ability in tiny pests sizes are far better than these object detection algorithms for each performance. On the “very tiny pest” test C, our model test results break 40% and 50% at P and mAP for the first time, reaching 40.9% and 57%, respectively, which is 6.6% and 28.5% higher than the second highest. Under the comparison from the mAP results in test C, our model is twice as high as the second highest, which is a huge improvement. This shows that our comprehensive performance is far superior to other object detection models while maintaining the highest accuracy. Meanwhile, F1 also showed the best results in both types of test sets, which also ensures the robustness of our model. It is worth noting that in the results of test C in **Table 5**, the R-value is too high in the performance of YOLOv9, while the P-value seems to be not as good. We have analyzed this, most likely because the adaptive anchoring box is not effective, there may be a result that causes the box to be larger for the object, but the information contained in the box increases as the box gets larger, thus resulting in too high and R-value and too low a P-value. The results of the B and C tests for the two types of pests show that our model is 4.1% and 32.2% higher in mAP than the baseline YOLOv8, respectively, which suggests that our improved method is very effective. Overall, the results prove that our method is the best when it comes to detecting “tiny pests” and “very tiny pests”. Our method not only results in new heights, but also far exceeds other models. Especially in the detection of “very tiny pests”, our mAP is twice as high as the second highest. Thus our method has the advantage of both high accuracy in these two types of pests detection and guarantees the robustness and stability of the model, which gives over help in agricultural pests detection.

**Table 5:** Test results for “tiny pests” and “very tiny pests”.

model	test B				test C			
	P(%)↑	R(%)↑	mAP(%)↑	F1↑	P(%)↑	R(%)↑	mAP(%)↑	F1↑
YOLOv3	65.8	89.6	77.5	0.76	34.3	43.2	22.6	0.38
YOLOv5	65.6	83.2	80.6	0.73	31.4	48.9	26.2	0.38
YOLOv7	57.2	72.9	70.5	0.64	31.3	55.8	28.5	0.4
YOLOv8	62.9	85.4	71.7	0.72	32.1	36.8	24.8	0.34
YOLOv9	68.4	85.8	83.6	0.76	25.4	**63.5**	25.3	0.36
SRNet-YOLO(ours)	**71.1**	**95.8**	**92.4**	**0.82**	**40.9**	42.3	**57**	**0.42**

The bold value means the highest value, and the symbol (↑) means the higher value is better.

### 5.5 Yellow sticky traps dataset (Low-resolution) validation results

Our study utilized the public the Yellow Sticky Traps dataset (low resolution) to evaluate the performance of our SRNet-YOLO model as shown in **Table 6**. This evaluation highlights the performance of our model compared to YOLOv3, YOLOv5, YOLOv7, YOLOv8 and YOLOv9 under low-resolution pest conditions. Our model still maintains the highest performance across metrics. Compared to the baseline YOLOV8, we were 1.1%, 1.7% and 0.7% higher in P, R and mAP, respectively. Because the Yellow Sticky Traps dataset was taken in a laboratory environment, the captured dataset is cleaner and clearer compared to the dataset we collected ourselves. Therefore, the detection accuracy of each model is higher compared to test A, B, C. Therefore, the improvement of our model in the detection of this dataset is not as high as that of the wild environment, and these situations are normal. Thus, the results confirm the advanced ability of SRNet-YOLO to maintain high detection accuracy with fewer pixels, emphasizing its suitability for applications in agricultural environments that require precise identification of microscopic pests, even in challenging low-resolution situations.

**Table 6:** Test results for the Yellow Sticky Traps (low-resolution) data.

model	test D (low-resolution image)			
	P(%)↑	R(%)↑	mAP(%)↑	F1↑
Yolov3	84.4	89.4	90.1	0.87
Yolov5	86.3	90.3	92.7	0.88
Yolov7	83	84.8	88.7	0.84
Yolov8	85.5	90.6	92.1	0.88
Yolov9	83	87.8	89.6	0.85
SRNet-YOLO(ours)	**86.6**	**92.3**	**92.8**	**0.89**

The bold value means the highest value, the symbol (↑) means the higher value is better.

### 5.6 Visualization and analysis

**Figure 9:**
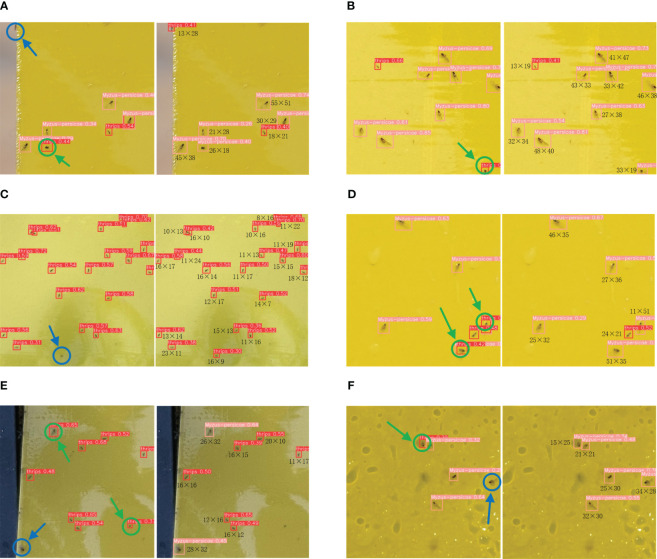
Comparison plots for visualization of cotton field count images. Where **(A–F)** are examples of different visualized image comparisons, the left side of each set of images **(A–F)** shows the detection results of YOLOv8, and the right side of each set of images **(A–F)** shows the detection results of SRNet-YOLO. Where green arrows represent false detections and blue arrows represent missed detections. We also labeled the pixel size of each pest in the detection results of the SRNet-YOLO model.

In the visual evaluation of pest detection algorithms on cotton field images, our SRNet-YOLO model’s performance is significantly superior to that of YOLOv8, as illustrated in **Figure 9**. Our method demonstrates enhanced precision, with a notable decrease in both false positives and false negatives, improving the accuracy of pest detection in agricultural applications.

The efficacy of our SRNet-YOLO is particularly evident in its ability to correctly identify pest by pixel size, as indicated in the results. This level of detail is crucial for precise pest population estimation, which is a key factor in effective pest management. Our approach, which reduces erroneous detections, provides valuable insights for developing targeted pest control strategies, ensuring that interventions are accurately aligned with the pest pressure indicated by the reliable detections. Confirming the findings of **Tables 5**, **6**, our model is especially adept at detecting smaller and less distinct pests, minimizing the risk of overlooking “tiny pests” and “very tiny pests” that may cause crop damage. The success of our model in challenging wild conditions emphasizes its potential to advance precision agriculture, enabling more accurate and efficient pest management.

## 6 Conclusion

In the pursuit of enhancing detection of tiny pests in wild natural cotton fields, this study has tackled the challenging task of identifying “tiny pests” and “very tiny pests”. We introduced a novel backbone network that integrates super-resolution reconstruction with the YOLOv8 framework, tailored for precise detection. We combined SPPCSPC with SRResNet and add jump connections to form a new feature map super-resolution reconstruction module. This significantly enhances feature recovery and identifies pests more accurately and in detail. To solve the problem of information loss after super-resolution reconstruction, we designed a feature fusion module based on BiFormer attention. This effectively preserves the feature of the pests and provides for the subsequent extraction of more distinct features, which greatly improves the detection accuracy and performance. To validate the performance of the model, we also built the dataset containing “tiny pests” and “very tiny pests” (Cotton-Yellow-Sticky-2023). Our experimental results demonstrated that compared to YOLOv3, YOLOv5, YOLOv7, YOLOv8 and YOLOv9, our method outperforms in mAP metrics by 6.9%, 7.2%, 5.7%, 4.1% and 5.6%, respectively. We also performed a more convinced experiment on “tiny pests” and “very tiny pests”. In the test of “tiny pests”, Our method has better performance compared to other model, with a mAP value of 92.4%, which is 20.7% higher than YOLOv8. In the “very tiny pests” test, our accuracy breaks through 40% and is the highest accuracy, while the mAP value reaches 57%, which is 28.5% higher than the second highest. Furthermore, when applied to the Yellow Sticky Traps dataset (low-resolution), our method not only outperforms YOLOv8 but does so with marked increases of 1.1% in Precision, 1.7% in Recall, 0.7% in mAP, solidifying its advantage across all metrics in challenging detection scenarios. Our contributions to the detection of tiny and very tiny pests in wild cotton fields have advanced the capabilities of one-stage object detection models, have laid the groundwork for automated crop pest diagnostics, enhancing real-time monitoring and management in agriculture.

## Data Availability

The original contributions presented in the study are included in the article/supplementary material, further inquiries can be directed to the corresponding author/s.
